# Supplementary figures and images for: Clu1/Clu form mitochondria-associated granules upon metabolic transitions and regulate mitochondrial protein translation via ribosome interactions
Source: PLoS Genet. 2025 Jul 7;21(7):e1011773. doi: 10.1371/journal.pgen.1011773 (PMC12262889; doi:10.1371/journal.pgen.1011773)

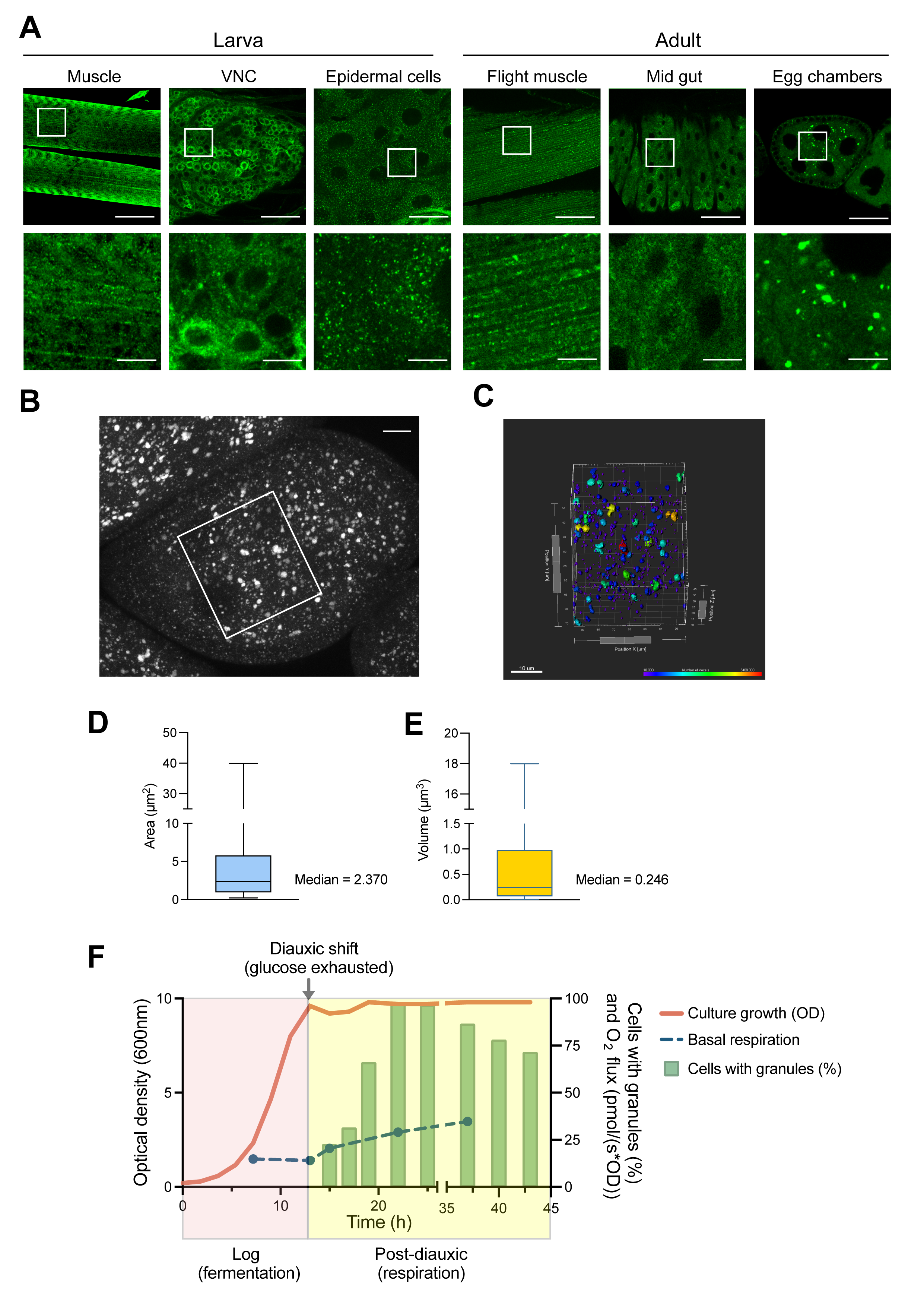

Supplement: S1 Fig — (A) Confocal imaging of GFP-Clu third instar larval and two-day-old adult tissues. VNC, ventral nerve cord. Scale bars: 40 µm (top panels) and 8 µm (bottom panels, magnified images of boxed areas). (B) Maximum intensity projection of an egg chamber of a three-day-old female fly refed for 6 h. The white box indicates the region used for 3D rendering in C. (D, E) Box plots representing the area (D) and volume (E) of Clu foci from the region used for 3D rendering. The plots display the minimum, first and third quartile, median and maximum values. (F) Graph showing Clu1-GFP cells’ growth in glucose-containing media, percentage of cells containing Clu1-GFP foci and basal respiration throughout growth. Cells undergo a diauxic shift upon exhaustion of glucose from the media. During this time, cells cease growth and suffer a drastic transcriptomic and proteomic shift to adapt to the new carbon source available in the media, ethanol. They transition from a fermentative to a respiratory metabolism. We considered the start of PD phase as the moment cells started regrowing and respiration increased. (TIF) [file pgen.1011773.s001.tif]

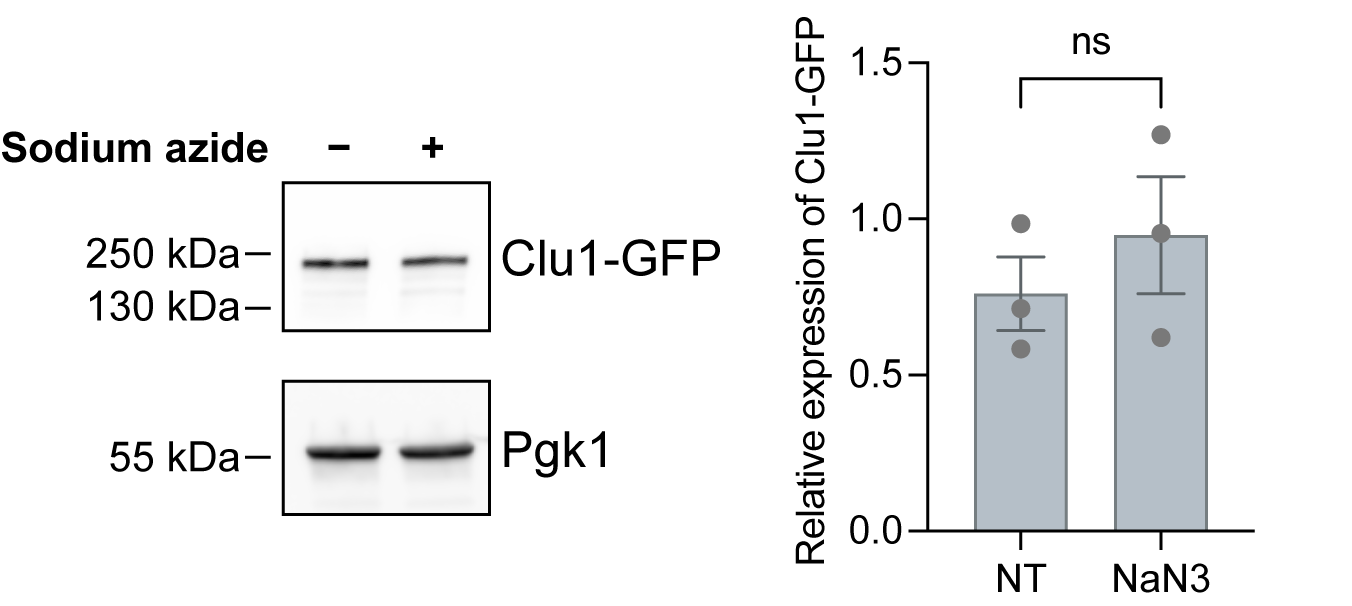

Supplement: S2 Fig — Immunoblot analysis of Clu1-GFP and Pgk1 in Clu1-GFP-expressing cells, either not treated (NT) or treated with sodium azide (NaN₃) to induce granule formation. The graph indicates the Clu1-GFP immunoblot quantification normalised by Pgk1 (mean ± SEM; n = 3; paired t-test; ns = non-significant). (TIF) [file pgen.1011773.s002.tif]

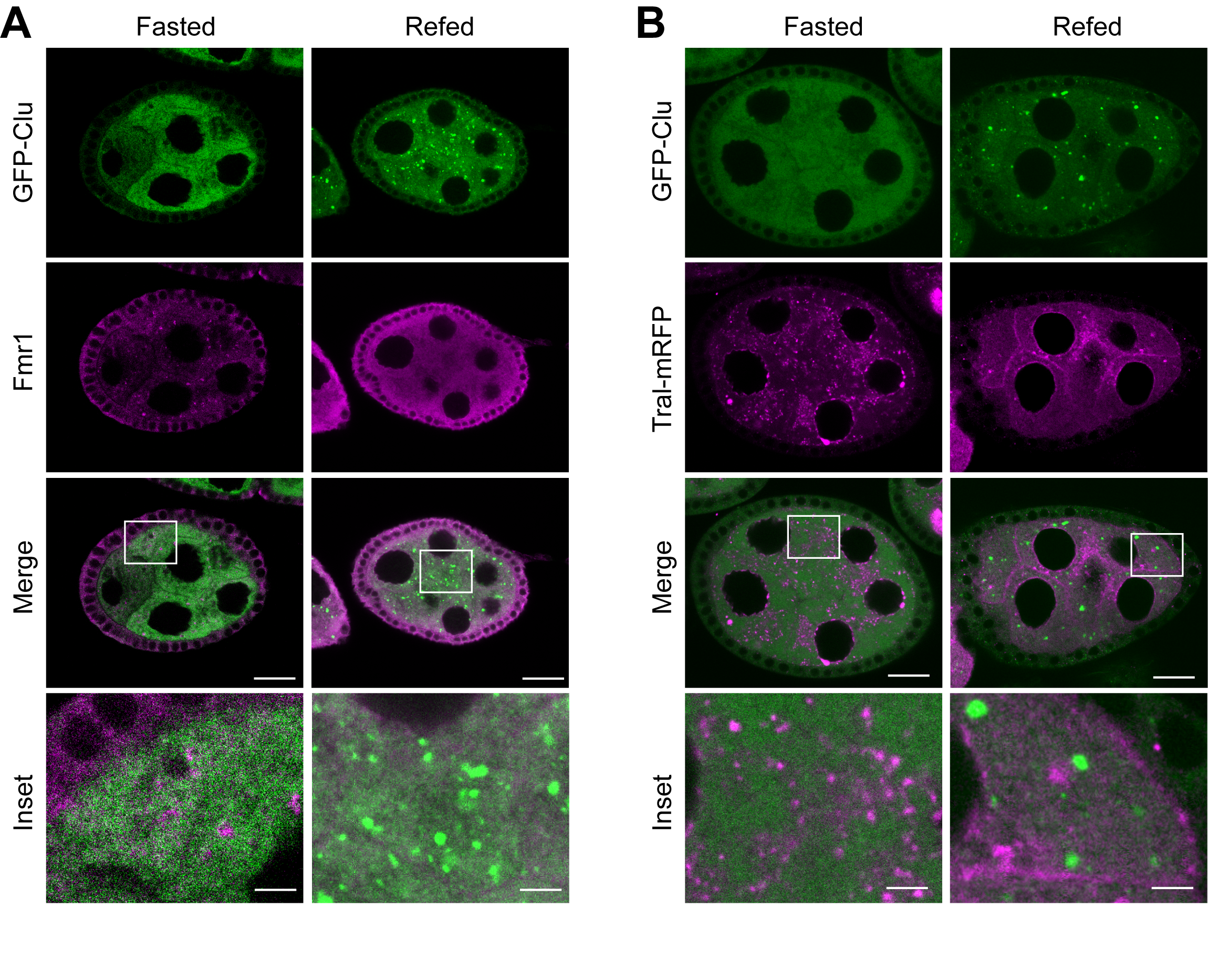

Supplement: S3 Fig — (A, B) Confocal microscopy of GFP-Clu egg chambers fasted (16 h) or refed (6 h) and immunostained for (A) Fmr1 (SG marker) or (B) co-expressing Tral-mRFP (PB marker). Scale bars = 20 µm, inset = 4 µm. (TIF) [file pgen.1011773.s003.tif]

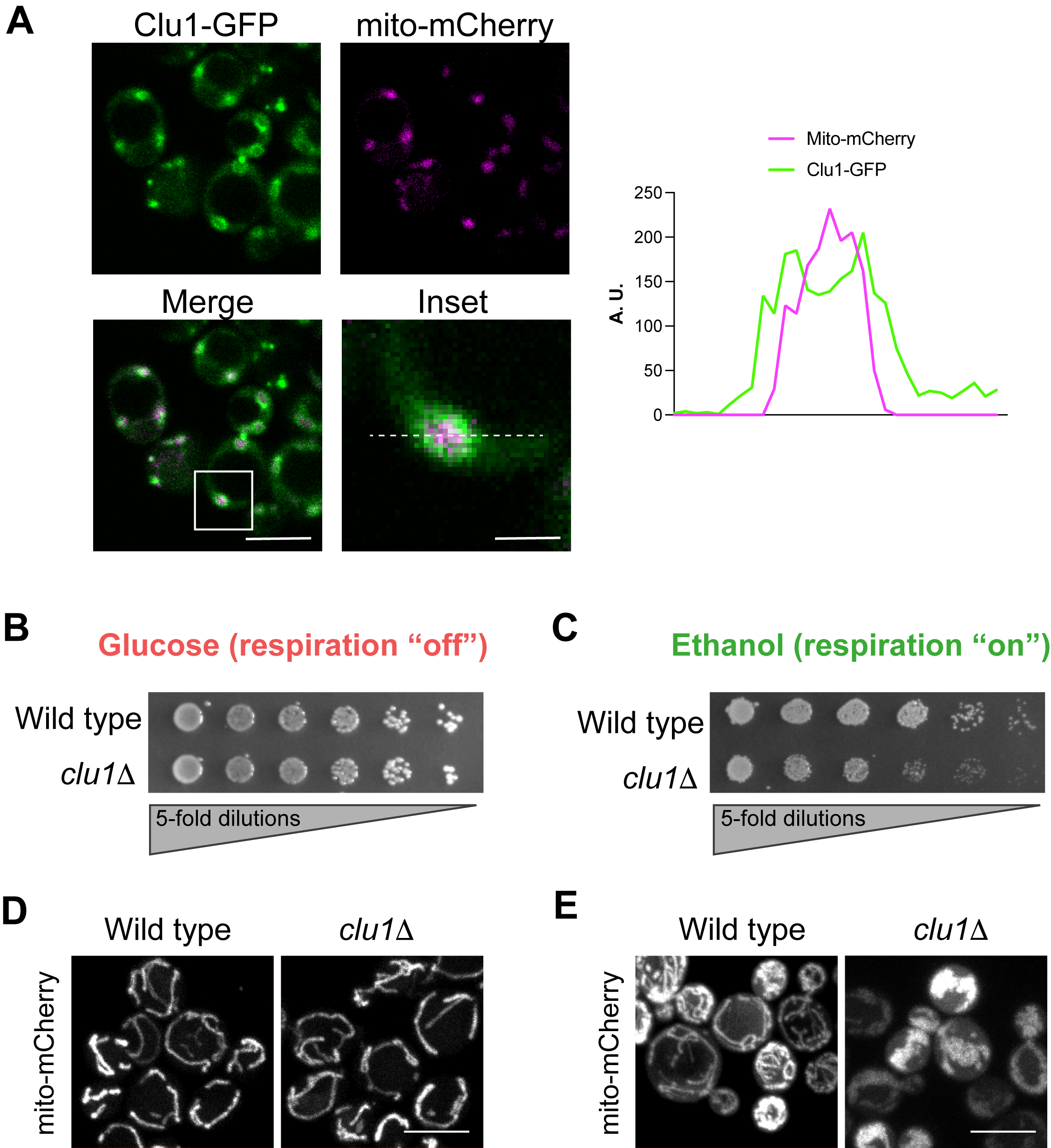

Supplement: S4 Fig — (A) Confocal image of Clu1-GFP cells expressing mito-mCherry in the PD phase. Inset box indicates zoomed image, with the intensity profile plot along the dashed line for Clu1-GFP and mito-mCherry fluorescence. (B, C) Spotting assay of clu1Δ and the parental strain W303A. Mid-log phase cells grown in glucose-containing media were adjusted to the same optical density, five-fold serial diluted and spotted onto media containing either glucose (B) or ethanol (C) as carbon sources. (D, E) clu1Δ and wild-type cells expressing mito-mCherry were imaged by confocal microscopy in media containing either glucose (D) or ethanol (E). Scale bars = 5 µm, inset = 1.25 µm. (TIF) [file pgen.1011773.s004.tif]

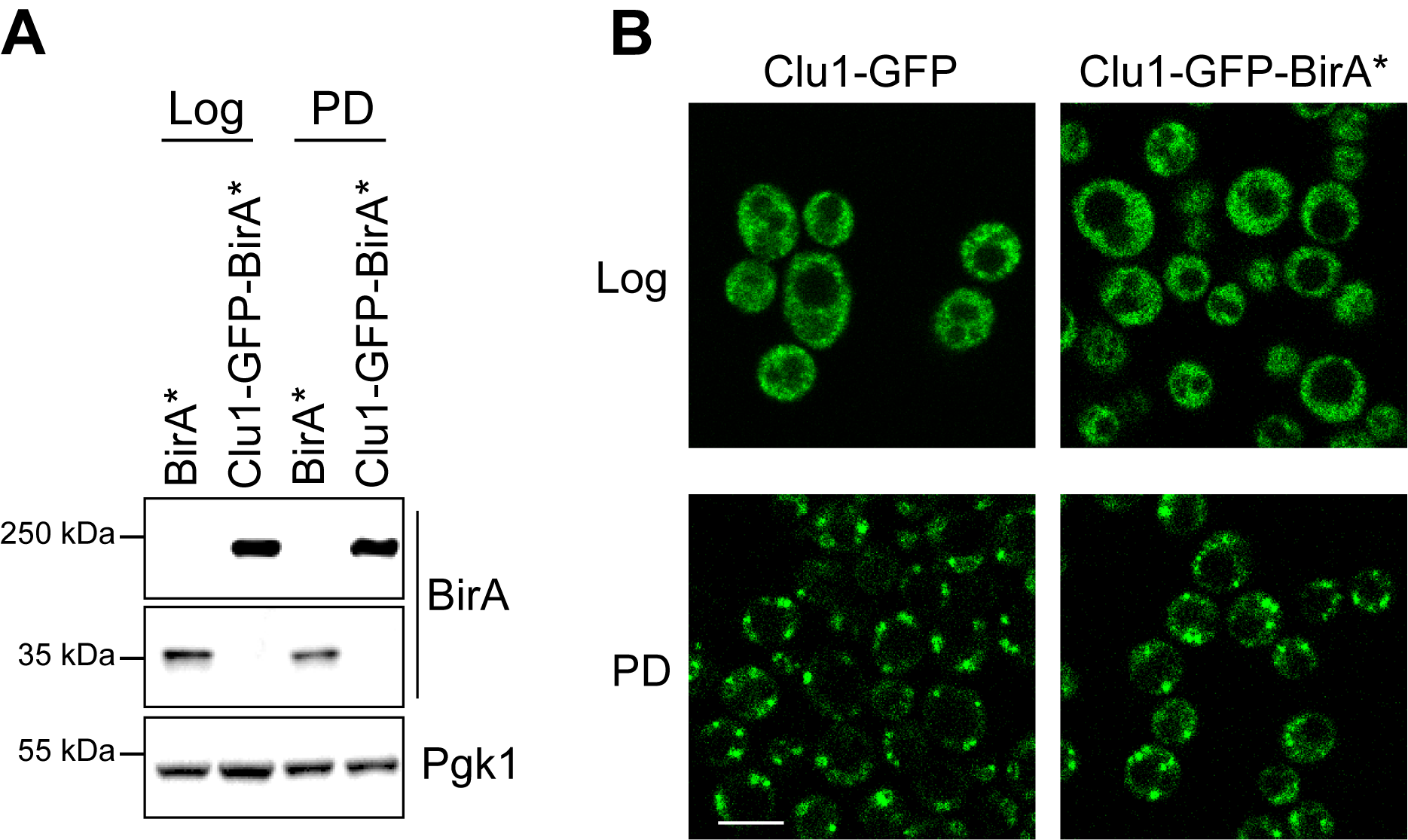

Supplement: S5 Fig — (A) Immunoblot of BirA* and Clu1-GFP-BirA* strains grown in the log and PD phases, with Pgk1 as loading control. (B) Confocal images of Clu1-GFP and Clu1-GFP-BirA* cells in the log and PD phases. Scale bar = 5 µm. (TIF) [file pgen.1011773.s005.tif]

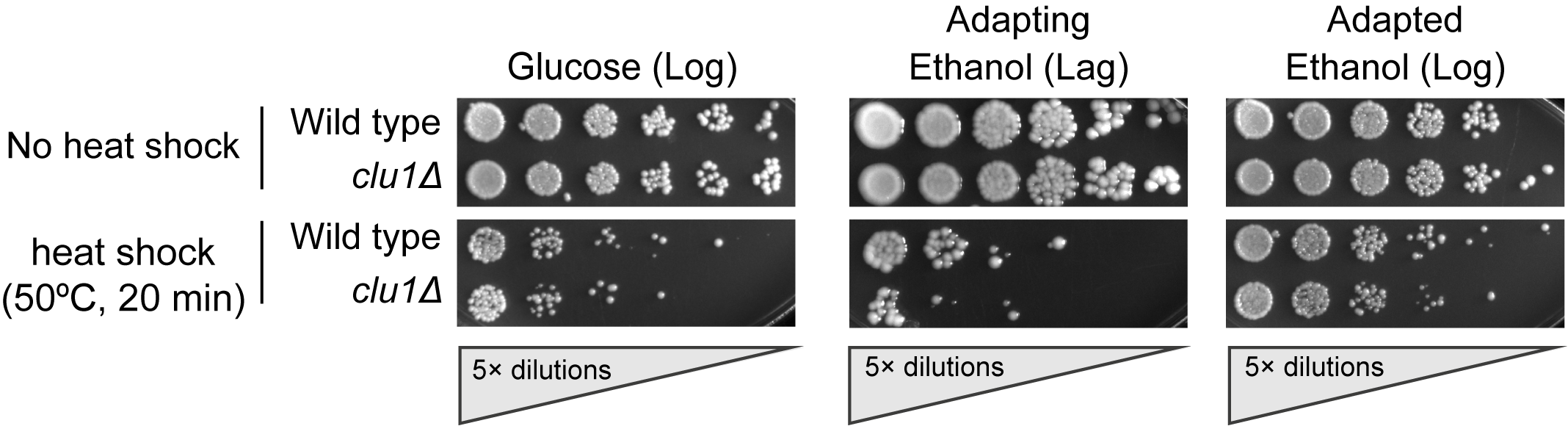

Supplement: S6 Fig — Spotting assay of clu1Δ and wild-type cells. Cultures were grown to early log phase in glucose-containing media at 30 ºC, then heat shocked at 50 ºC for 20 minutes or left at 30 ºC (no heat shock), under three conditions: from original growth conditions (Glucose (log)), following a shift to ethanol-containing media during the lag phase while cells were adapting to respiration (Ethanol (lag)), or after cells had resumed growth (Ethanol (log)). Cultures were normalised to the same optical density, serial diluted, and spotted onto glucose-containing media to assess survival. (TIF) [file pgen.1011773.s006.tif]

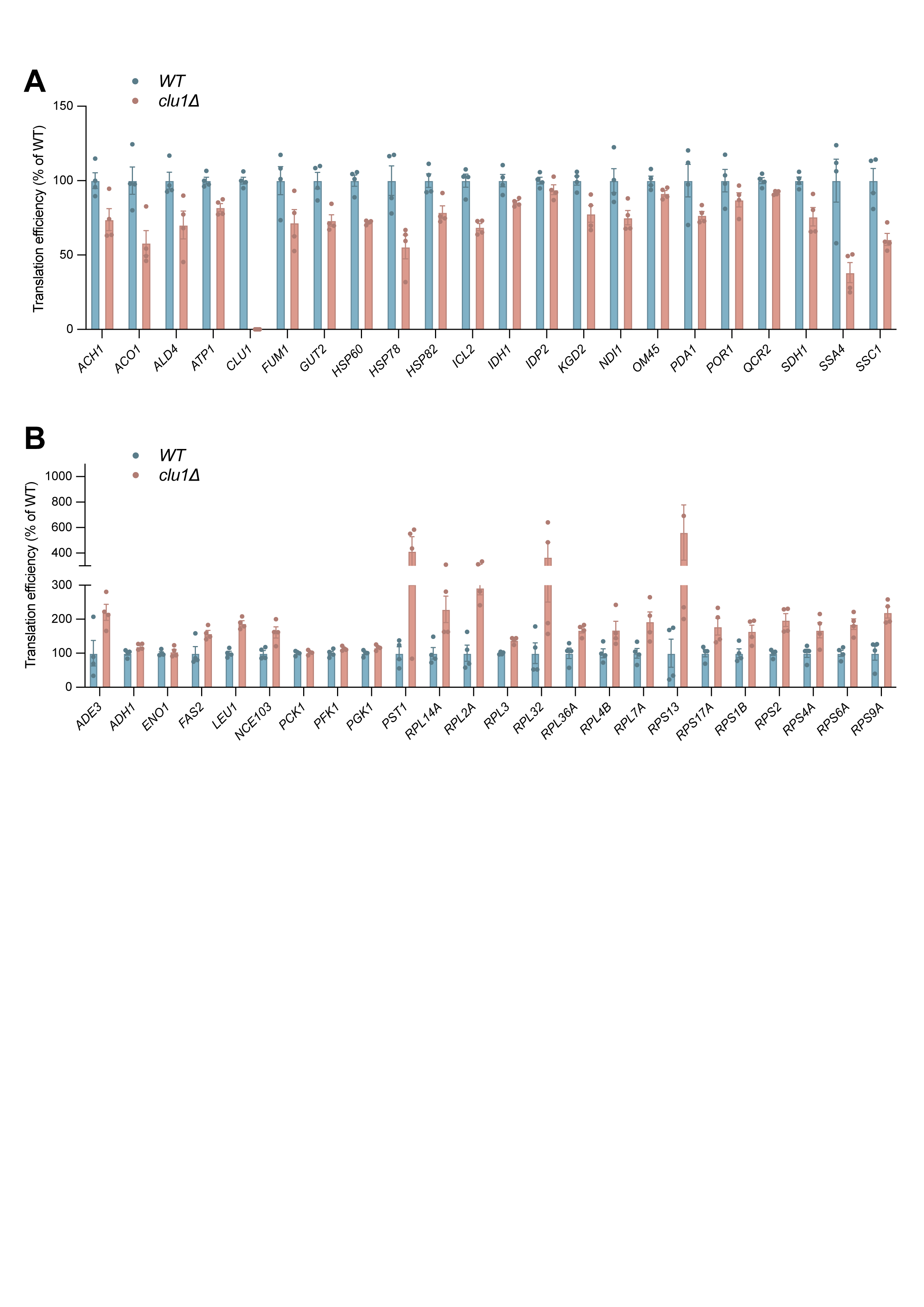

Supplement: S7 Fig — Graphs indicate the translation efficiency for mRNAs whose translation was reduced (A) or increased (B) in clu1Δ strain compared to wild type. Translation efficiency was calculated as the ratio of nascent protein levels (Punch-P) to the steady-state levels of their corresponding mRNA (RNAseq) and normalised to the wild-type levels. (TIF) [file pgen.1011773.s007.tif]
